# The Transition State of PBLG Studied by Deuterium NMR

**DOI:** 10.3390/polym17243280

**Published:** 2025-12-10

**Authors:** Fabian M. Hoffmann, Burkhard Luy

**Affiliations:** Institute for Biological Interfaces 4 (IBG-4), Karlsruhe Institute of Technology (KIT), Kaiserstrasse 12, 76131 Karlsruhe, Germany; fabian.hoffmann@kit.edu

**Keywords:** PBLG, poly-γ-benzyl-L-glutamate, transition state, biphasic region, kinetics, phase transition, quadrupolar couplings, NMR, partial alignment, deuterium

## Abstract

The liquid crystal (LC) poly-γ-benzyl-L-glutamate (PBLG) is known to possess a narrow biphasic range at the phase transition from an isotropic liquid to an anisotropic liquid crystal. We have characterized the biphasic region via deuterium nuclear magnetic resonance (NMR) of the deuterated solvent CDCl_3_, with which isotropic and anisotropic populations can unambiguously be identified and quantified due to the quadrupolar coupling induced by partial alignment. In addition to a dilution series, we measured the kinetics of the alignment inside the magnet for each dilution step and were able to follow the kinetic buildup of partial alignment. Beginning with the dynamic line broadening indicative of slow fluctuations, to microheterogeneous patches of isotropic and anisotropic islands, with increasing island size being consistent with sharpened spectra, ending in fully separated isotropic and anisotropic phases on top of each other after two weeks. In addition, we studied the influence of the two example guest molecules borneol and camphor—which essentially differ in their capability to act as hydrogen bond donors or acceptors—on the biphasic region of PBLG.

## 1. Introduction

PBLG as a chiral liquid crystal aligns in a magnetic field of sufficient strength above a certain concentration and induces anisotropy [1,2,3]. This effect has been used extensively for the measurement of residual anisotropic NMR parameters like residual dipolar couplings, residual quadrupolar couplings, and residual chemical shift anisotropies of solute molecules dissolved in the lyotropic mesophase with an organic solvent, typically deuterated chloroform (CDCl_3_). A vast number of structure refinements [4,5,6,7] and determinations of enantiomeric excess [8,9,10,11] are reported. But the residual anisotropic parameters can also be used to learn something about the constitution of the polymeric phase. In particular, the deuterated NMR solvent can be used as a spy solvent to detect aligning properties of the polymeric phase. For static samples, the alignment director occurs in the z-direction, the direction of the large static magnetic field used in NMR spectroscopy to induce measurable polarization. As we showed in an earlier publication, it is also possible to rotate this direction close to the x,y-plane by using shear forces [12]. We also found that below the critical concentration of the liquid crystalline phase, the seemingly isotropic phase can be forced into anisotropy. During these measurements, we observed a previously reported biphasic concentration range, which is common for PBLG and other liquid crystals [13,14,15,16,17] and is theoretically described by Flory [18]. Under shear force, this coexistence of isotropic and anisotropic phases transforms into a homogeneously anisotropic one [12]. The concentration range of the observed biphasic phase varies a lot with temperature, the solvent used, and the rod length. At ambient temperature, the PBLG solvent mixture is biphasic only in a small region. Below this “chimney” region, a wide biphasic area can be found for lower temperatures [15,16,17,19,20]. Flory [18] predicted the concentration of rodlike polymer mixtures in a chimney between 8/x and 12.5/x with the aspect ratio x = L/D. L and D are hereby the length and diameter of the polymer rods. Miller et al. [21] found different PBLG concentration ranges for the chimney regions of different solvents. For DMF it was biphasic between 8.5% and 11.5%, methylene chloride between 8.0 ± 0.5%, and 12–15% and dioxane between 7.0 ± 0.5% and 9–11% for different lengths of PBLG. In particular, the rod length changes the appearance of the biphasic region dramatically, as Wu et al. [20] showed in corresponding phase diagrams for PBLG.

While ^1^H and ^13^C NMR spectroscopy of the polymer becomes very difficult in an aligned state with tremendously broadened signal widths, liquid crystalline phases and their phase transitions can generally be characterized very well by deuterium NMR spectroscopy of the solvent [22,23]. In an isotropic environment, the solvent signal of CDCl_3_ is a singlet, as all anisotropic parameters (e.g., dipolar and quadrupolar couplings) cancel each other, with no effective splitting being observed. A liquid crystalline phase is usually aligned by the strong magnetic field present in a conventional NMR spectrometer, which leads to an anisotropic environment for solvent and solute molecules. In an anisotropic environment, on the other hand, coupling Hamiltonians do not average to zero, but to an averaged value, so-called residual couplings. The effect is clearly seen with deuterium nuclei with spin *I* = 1 and correspondingly strong quadrupolar couplings Q. Residual quadrupolar couplings manifest into a doublet in deuterium spectra, where the observed splitting is proportional to the averaged strength of the alignment experienced by the partially aligned solvent molecule [24,25]. In the special case of a biphasic region, we can see both the isotropic peak at the standard chemical shift of the solvent and the two anisotropic peaks with ±Q/2, where the height and integrals of all three peaks change with concentration and temperature [12,23]. Here, we use deuterium NMR spectroscopy to characterize details of the isotropic-to-anisotropic phase transition with dilution series and to monitor the development of liquid crystalline regions in kinetic studies.

**Figure 1:**
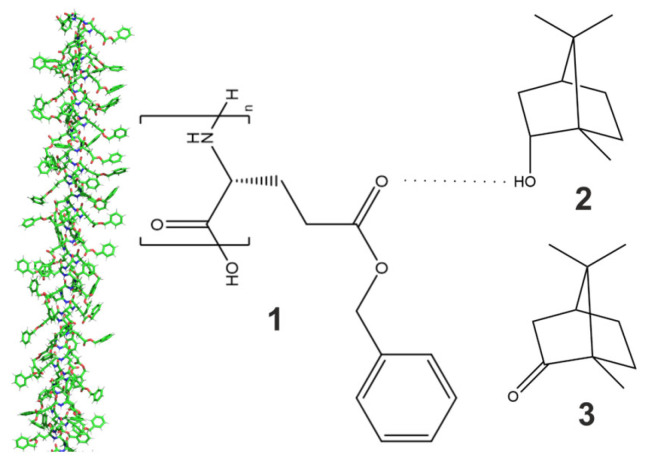
The liquid crystal PBLG (**1**) can form hydrogen bonds with its acceptor groups at the free ester carbonyl of the side-chain. In the backbone, α-helical PBLG forms intramolecular hydrogen bonds. In this work, borneol (**2**) is used as a potential hydrogen bond donor to PBLG, and camphor (**3**) as a hydrogen bond acceptor solute molecule unable to form hydrogen bonds with PBLG.

In a previous study using PBLG as a so-called alignment medium [12], we found an influence of solute molecules on the appearance of the biphasic region. These results were corroborated by theoretical findings [26] concerning solute–polymer interactions with PBLG and specific chiral molecules [27]. We therefore also added borneol and camphor to PBLG (Figure 1) in dilution series as two previously used molecules that essentially differ in their hydrogen bond capability to find out about the influence of weak binding processes.

## 2. Materials and Methods

### 2.1. Preparation of the PBLG Samples

All samples were prepared by weight percentage of PBLG (mol wt 150,000–350,000 purchased from Merck, Darmstadt, Germany) and chloroform-d_1_ (used without further purification and stored with silver foil), as earlier published by Lesot et al. [1]. The addition of (+)-borneol and (−)-camphor, with 14 mg of each, follows the same recommendation. The two samples with borneol and camphor, as well as one sample without a guest molecule, were all prepared from the same batch. A second sample (Section 3.2) without a guest molecule was from a different batch and resulted in slightly different alignment properties. For the dilution steps, CDCl_3_ was added each time by weight. All PBLG samples were directly prepared in the NMR tube and afterwards thoroughly centrifuged up and down approximately 20 times until the sample was fully homogenized. After each dilution step with CDCl_3_, the samples were likewise centrifuged.

### 2.2. NMR Measurements

Single-scan 1D ^1^H and 1D ^2^H spectra as well as 2D ^2^H image spectra [28] were acquired at 300 K on a 600 MHz Bruker Avance III HD spectrometer with a BBI room temperature probehead (Bruker BioSpin GmbH, Rheinstetten, Germany). ^1^H spectra at 600.19 MHz were measured with 32k complex data points, and ^2^H spectra at 92.13 MHz with 8k complex data points in the direct dimension. Deuterium images were taken with 8k complex data points and one scan in the direct dimension and 64 increments with increased gradient strengths in the indirect dimension.

The process of placing the sample into the magnet and starting the first experiment (including the ~2 s acquisition + delay) took approximately 5 s. For the first 60 s, every 5 s, a spectrum was taken with the same sample. As the changes were minor after the first 20 s, the next time points were chosen as ~4, 8, 10, 15, 20, 30, 40, 50, and 60 min. Except for the concentrations around 50:50 iso/aniso, these intervals were sufficient. Longer intervals (100 min and more) were only conducted around the latter concentrations. Besides the estimated initial injection period, further intervals were obtained via defined relaxation periods in corresponding pulse programs.

## 3. Results

All following studies involving NMR spectra were conducted via deuterium NMR spectra of the solvent signal of deuterated chloroform, whose isotropic peak has a chemical shift of 7.26 ppm. All experiments were performed on a 14.1 T/600 MHz NMR spectrometer with a deuterium Larmor frequency of 92.1 MHz. For liquid crystalline PBLG in CDCl_3_, the peak is split by a residual quadrupolar coupling Q due to the resulting anisotropic environment. Therefore, during a biphasic transition, three peaks are visible, defining the isotropic subphase by the singlet and any partially aligning liquid crystalline phase by the surrounding doublet. Relative integral ratios of the two signals are indicative of the concentrations of the subphases, and signal heights are a measure of the homogeneity of the environment felt by solvent molecules over the course of the experiment.

### 3.1. Dilution Study Without Solute Molecules

**Figure 2:**
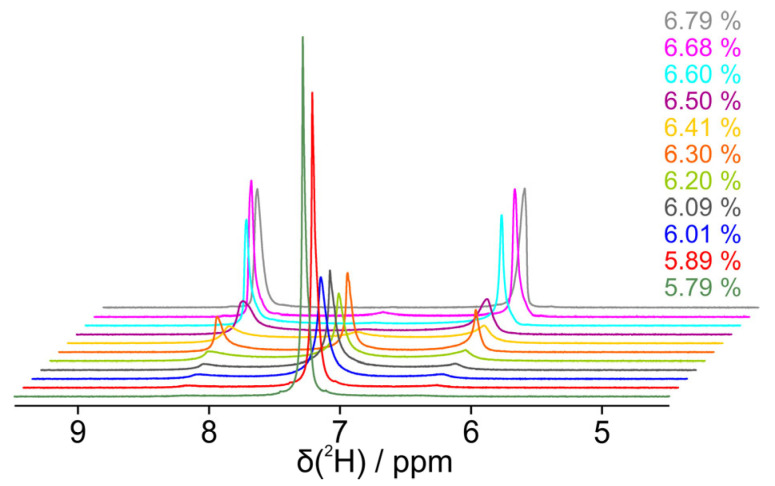
The isotropic liquid to anisotropic liquid crystal phase transition of PBLG/CDCl_3_ studied by deuterium NMR of the solvent. Deuterated chloroform was added to PBLG during a step-by-step dilution from a pure quadrupolar split (anisotropic) signal (188 Hz corresponds to ~2.04 ppm, resulting in signals at 6.24 and 8.28 ppm) to a single (isotropic) signal (7.26 ppm). Spectra were taken 5–10 min after the freshly diluted and centrifuged sample was inserted into the magnet.

In an initial dilution series, a sample without a guest molecule started at a concentration of 10.26% PBLG in CDCl_3_ with a deuterium quadrupolar splitting of 378 Hz and was titrated with solvent in coarse steps of approximately 0.2% until a concentration of 6.79% and a quadrupolar splitting of 188 Hz was reached. The concentration was thereby determined by the relative mass of PBLG and CDCl_3_. Dry PBLG was weighed in the beginning in the NMR tube, before CDCl_3_ was added. The total weight of the NMR tube with unchanged amounts of PBLG and the differing amounts of CDCl_3_ was then determined at every stage of the dilution series. The quadrupolar splitting in the initial concentration range decreased linearly, as reported previously [24,25]. Below a 6.79% PBLG concentration, a small singlet appeared in the center of the split signal of the deuterated solvent, indicating an isotropic component in a biphasic system (Figure 2). With further, finer dilution, the isotropic peak increased and the outer anisotropic peaks decreased. At a concentration of 6.50%, the inner peak was still small, but the outer peaks broadened significantly, leading to a type of coalesced spectrum at 6.41% concentration. With further dilution, peaks sharpened significantly with a more and more dominating isotropic component. At a concentration of 5.79%, only the central isotropic signal is visible. For all measured concentrations, the boundary of the quadrupolar coupling stayed constant at 186 ± 2 Hz.

### 3.2. Measurement of Kinetic Behavior of Coexisting Phases at the Transition

**Figure 3:**
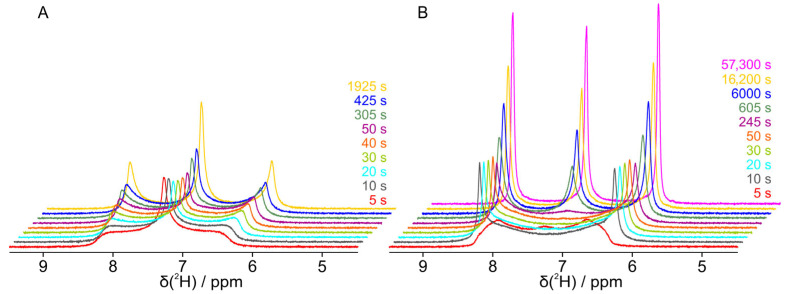
Kinetic studies of PBLG/CDCl_3_ samples for two different concentrations within the biphasic region. (**A**) At a concentration of 6.03% wt PBLG, a *f_ani_* of 53% is obtained. While anisotropic (two outer lines) and isotropic signals (central lines) show roughly identical overall integrals, the intensities are steadily increasing. We stopped the experiment when the areas between peaks reached the baseline after approximately 40 min. (**B**) The same effect is seen for a sample at 6.19% wt concentration with roughly twice the integral of the anisotropic compared to the isotropic component, corresponding to a *f_ani_* of 65%. For an explanation of the exact measurement procedure for anisotropic fractions, please look at Figure 4.

The observed changes in linewidths in the dilution series led us to the assumption that the solute ^2^H-NMR spectra might be sensitive to kinetics at the transition from the pure anisotropic to the pure isotropic phase. Both pure phases take less than a minute to fully equilibrate in our 14.1 T magnet, as we could verify by deuterium spectra. Within the biphasic region, however, slower dynamics appear, which we would like to characterize in the following section. For a practical description, we thereby define the anisotropic fraction *f_ani_* as

(1) $$f_{ani}=\frac{I_{ani}}{\left(I_{ani}+I_{iso}\right)}$$

with *I_ani_* and *I_iso_* corresponding to the integrals of the anisotropic doublet and the isotropic singlet, respectively. Below an anisotropic fraction *f_ani_* of 5% and above 95%, it takes less than a minute to see no further changes in the spectra. Between an *f_ani_* of 5% to 20% as well as 80% to 95%, equilibration is reached in less than 20 min. After 20 min, only minute changes in spectra and integrals were observed for the baseline-separated peaks. In the *f_ani_* ranges of 20–40% and 60–80%, the process slowed down to 30–40 min, and for *f_ani_* of 40–60% more than 100 min were needed until baseline separation was reached. Remarkably, around a *f_ani_* of 50%, signals still sharpened noticeably after 4 h and even after half a day, slight changes could be seen before we stopped our measurements (see Figure 3 for spectra of two example concentrations).

**Figure 4:**
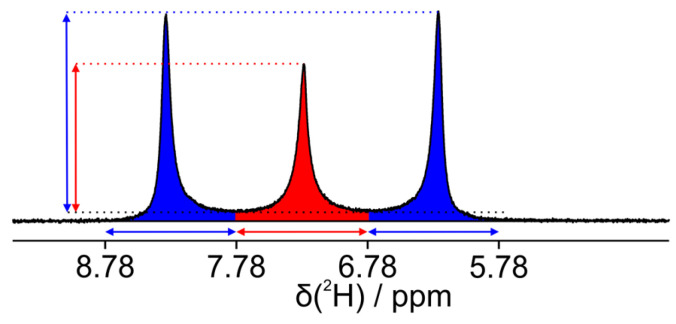
Explanatory scheme for measured signal heights and integrals. Blue shows the signal height and integral of the outer (anisotropic) peaks and red shows the same for the inner (isotropic) peak. Red areas are proportional to *I_iso_* and the combined blue areas represent *I_ani_*. For comparability reasons, the three integral regions were always chosen to be exactly 92.13 Hz (1 ppm), starting at 5.78 ppm.

**Figure 5:**
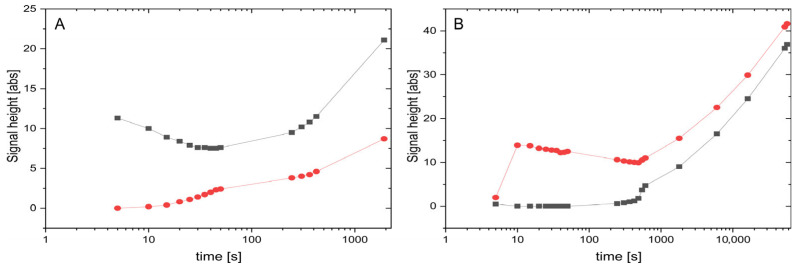
The time-dependent inner isotropic (black) and the outer anisotropic (red) signal heights for (**A**) the 6.03 wt% sample and (**B**) the 6.19 wt% sample, respectively. Note that lines still sharpen at the end of the observed timeslot.

Next to the integral fractions, as explained in Figure 4, we also measured the maximum signal heights of the isotropic inner line and the two anisotropic outer lines of the deuterium spectra and plotted them for the two example samples in Figure 5. The signal heights are indicative of the homogeneity of environments experienced by the individual molecules. Broad lines refer to molecules experiencing different amounts of isotropic and anisotropic subphases during the course of the NMR experiment (time scale of ~1 s). This is caused by Brownian motion (translational diffusion). Sharp lines with large signal heights, on the other hand, indicate that molecules—despite diffusion—experience only a uniform subphase. Rising signal intensities over time are therefore evidence of slowly growing clusters, separating the originally microheterogeneous clusters into larger and larger clusters of the subphases. For the sample at 6.19 wt% (Figure 3B and Figure 5B), the phase needs around 10 s to show the first humps as peaks out of the broad coalesced signal. Afterwards, the signal heights of the outer anisotropic peaks decrease slowly for nearly 500 s, probably due to technical reasons as the contribution of the heavily broadened inner line to the outer lines is more and more reduced with sharpening signals. As soon as the inner peak becomes visible and increases, all line intensities steadily grow for the sharpening signals. This is some kind of ‘turnover’ point where both peaks begin to increase. The sample at 6.03 wt% behaves similarly. Here, the dominant phase is the isotropic one and the inner peak decreases for the first 50 s, during which the severely broadened outer signals contribute less and less to the central peak. After the onset of signal separation, both peaks increase steadily for the rest of the 2000 s of observation, showing clear evidence of continuous separation of the isotropic and anisotropic subphases.

### 3.3. Influence of Borneol and Camphor on Phase Transition

**Figure 6:**
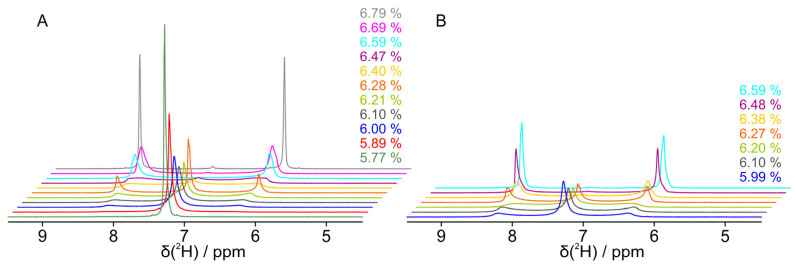
The transition of the deuterium spectrum of deuterated chloroform with PBLG and a guest molecule during step-by-step dilution from a pure quadrupolar split (anisotropic) doublet to a (isotropic) singlet. Spectra were acquired approximately 5–10 min after the sample was inserted into the magnet. The guest molecules are (**A**) borneol and (**B**) camphor.

Based on the experience of previous studies [26,27], we expanded the dilution study of pure PBLG/CDCl_3_ by two more samples, each with a different solute molecule. Camphor is a naturally occurring bicyclic ketone that can act as a hydrogen bond acceptor with its C=O bond. By reducing this ketone to an alcohol, we obtain borneol, another naturally occurring molecule that now acts as a hydrogen bond donor. As can be seen in Figure 6, the addition of these molecules influences the biphasic concentration range. With borneol, the first appearance of the isotropic middle peaks is at 6.79% as in the case without a guest molecule, but the outer peaks decrease faster and the turnover point of the titration, where all three peaks are of the same height, is already 6.47% compared to 6.41%, which is outside the expected error of the concentration determination. Also, with borneol, the outer peaks vanish at higher concentrations compared to the sample without a guest molecule. With camphor as the guest molecule, on the other hand, the first appearance of the central singlet is at a concentration of 6.59%, which is 0.2% less than for the other samples. Also, the titration turnover point at 6.27% is significantly shifted.

**Figure 7:**
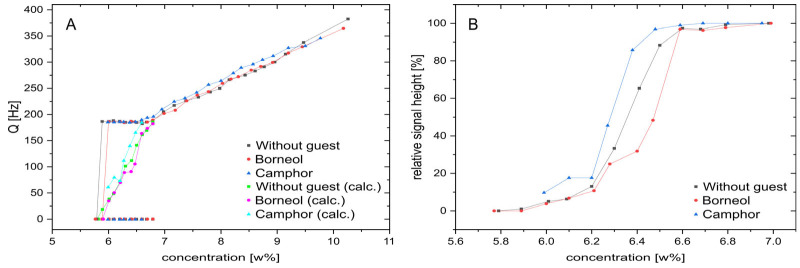
(**A**) Quadrupolar couplings of the three PBLG samples from the same batch for different concentrations. When the biphasic region is reached (Q ≈ 185 Hz), the additional 0 Hz coupling of the isotropic peak is included as well as the integral-weighted average couplings according to Equation (1). Please note the change in slope at purely anisotropic concentrations above ~6.8 wt%. (**B**) A zoomed view of the relative signal heights of the outer anisotropic doublet compared to the central isotropic singlet for the same three samples as in (A) to focus on the details at concentrations at the phase transition. Small but distinct effects of the solute molecules are visible.

In Figure 7, coupling and intensity data for the dilution series are extracted and in addition to the directly measured splittings in the biphasic region at the transition, the integral-weighted average coupling *Q_w_* is also calculated using the following formula:

(2) $$Q_{w}=f_{ani}Q$$

where *Q* represents the essentially constant observed splitting of the doublet of 185 Hz. If we compare the development of quadrupolar couplings of the deuterated chloroform signals for all three samples at all concentrations measured, the camphor sample always shows a slightly larger coupling compared to the other two samples. The latter ones, on the other hand, display very similar concentration dependencies and only differ in the biphasic concentration region. During the biphasic transition region, the development of the relative signal heights and the integral-weighted average couplings *Q_w_,* as calculated from Equation (2), borneol shifts the transition by +0.1 wt% and reaches the pure isotropic phase at higher concentrations. The sample with camphor, instead, shows a shift in the transition of −0.2 wt% relative to the pure PBLG/CDCl_3_ sample. The integral-weighted average coupling also indicates that the overall anisotropy decreases approximately linearly with decreasing concentrations in the fully anisotropic concentration region as well as during the biphasic transition state, although with different rates. This observation corroborates previous results with a rheological NMR device [12], where homogenization by shear forces displayed a similar behavior. Interestingly, the rates seem to be identical for the three samples studied; just a shift in the onset is observed.

## 4. Discussion

While we could not measure a biphasic transition state for PBLG/CDCl_3_ without solute molecules in a previous study [12], the detailed study shown here unambiguously proves the coexistence of isotropic and anisotropic microheterogeneous phases in PBLG/CDCl_3_ without any solute molecule at a 600 MHz NMR spectrometer. The data supports previous reports [19,20,21,29,30,31], but in addition, adds a quantitative, kinetic, and concentration perspective. We found that the actual concentrations at which the biphasic transition state is observed depend very much on the specific constitution of the polymer. Although in all studies we used the very same polymer from the same source (Merck, P5136-1G, Batch Number 0000368680), two different batches resulted in shifts in the transition concentration of approximately 0.5% wt. It was therefore crucial to perform all relevant measurements, like the dilution series with solute molecules, with the identical batch. We also took care to very carefully determine sample concentrations by exact weighing of the polymer and the amount of solvent and solute being added, resulting in estimated errors of concentration measurements on the order of 0.01% wt.

In all cases, the observed quadrupolar coupling of chloroform stays essentially constant over the entire biphasic concentration range and only peak integrals and intensities of the subphases change. An important result is therefore that the anisotropic phase does not change its characteristics but that only the proportions between the isotropic and anisotropic subphases change with concentration. This is in good agreement with the findings of Robinson [13,14], who showed in polarized images that the birefringent phase contains either numerous small monocrystals (spherulites) in the isotropic solvent or a continuous phase of these spherulites melted together.

**Figure 8:**
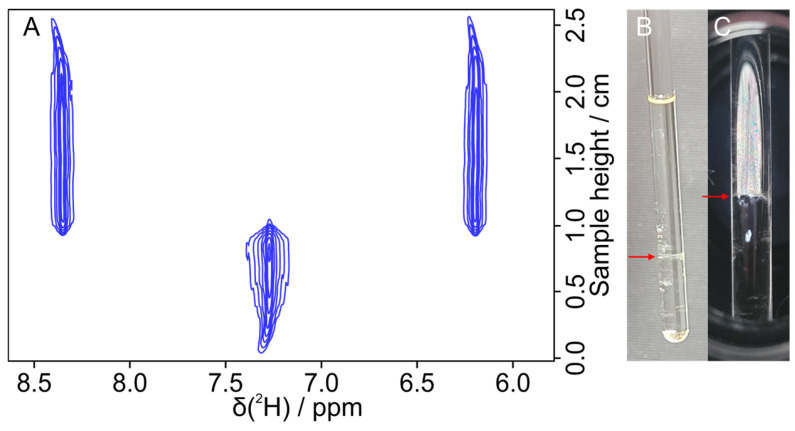
(**A**) Two-dimensional ^2^H image spectrum of a 7.0 wt% PBLG/CDCl_3_ sample. The image was taken 5 weeks after the last centrifugation, continuously stored on top of the NMR magnet with a constant stray field. During these 5 weeks, the sample was measured several times inside the magnet, with a total of 2 weeks of measurement time. (**B**) Picture of the separated sample under normal light. (**C**) Picture of the same sample outside the magnetic field under polarized light with both filters 180° to each other, filtering out the incoming light. The red arrows show the phase boundary.

The kinetic measurements with corresponding lineshape analysis shed more light on the microscopic details. Lines are very broad, without contours, right after inserting the sample into the magnet. At this stage, an almost heterogeneous sample forms islands of partially orienting polymer clusters and the detected solvent molecules experience statistically distributed amounts of oriented as well as isotropic patches. As small molecules at room temperature typically travel a distance of about 100 µm during the course of an acquired FID of approximately 1 s, cluster or spherulite size must have diameters significantly smaller than this distance. Over time, individual peak widths become sharper and sharper, indicating that solvent molecules are more and more confined to either only isotropic or only anisotropic environments. From this, we can draw the conclusion that islands of anisotropy increase over time, with more and more clusters exceeding a diameter of 100 µm. Proposed spherulites, therefore, must melt together over the course of seconds to hours to form larger and larger clusters. The process also continues on a longer time scale, eventually leading to complete isotropic and anisotropic phase separation over the course of weeks for the samples under study. Figure 8 shows a sample after 5 weeks, where the full separation of the upper aligned and lower isotropic phase can be observed optically as well as with a ^2^H-imaging experiment [28]. According to the literature [30,32], phase separation is possible if the densities of the isotropic phase and the slightly heavier anisotropic phase are sufficiently different. In the reported cases, the isotropic phase was always found to be at the top. Apparently, due to the high density of chloroform, the isotropic phase as the heavier one is found at the bottom of the NMR tube.

The differences in line-sharpening for the various anisotropic fractions would naively be interpreted as differing clustering speeds at different concentrations. The lineshapes, however, are given by dynamic exchange processes, which are known to be strongest at coalescence conditions, involving the exchange of signals with equal signal heights, as observed here (Figure 3B). We did not try a (rather difficult) lineshape analysis for the studied system, so we cannot make any statement about changes in clustering rates and have to state that the cluster formation process may well occur at a single rate independent of the concentration.

A comparison of the dilution series without a guest molecule with two other dilution series, one with a hydrogen bond acceptor and one with a hydrogen bond donor, shows that low amounts of solute molecules have an influence on the alignment and concentration behavior of PBLG. A hydrogen bond donor like borneol increases the necessary PBLG concentration for the transition and a hydrogen bond acceptor like camphor reduces it. As has been shown in previous studies, hydrogen bond formation of solute molecules with the ester carbonyls of the PBLG sidechain as hydrogen bond acceptors plays an important role in partial alignment of solute molecules. Borneol is known to interact relatively tightly with PBLG in a hydrophobic solvent like CDCl_3_, while camphor, without the possibility to form hydrogen bonds with PBLG, does not. If we assume that borneol is mostly bound to the PBLG side chains, it may influence the PBLG helix–helix interaction, which most certainly is based on the close aromatic interactions of the benzyl groups. Mostly aliphatic and bulky borneol, on the other hand, reduces this interaction, as borneol molecules first have to be displaced before an interaction can happen. A shift in the transition towards higher concentrations is therefore a logical consequence. Camphor, on the other hand, is not able to interact closely with the PBLG sidechain. It may, however, bind tightly to solvent molecules, thereby effectively reducing the amount of readily accessible CDCl_3_ for PBLG and explaining the lowered phase transition concentration.

## 5. Conclusions

In summary, a detailed analysis of the transition from isotropic to anisotropic liquid crystalline state of PBLG in CDCl_3_ is given with deuterium NMR spectroscopy of the deuterated solvent. The microheterogeneous co-existence of isotropic and anisotropically aligned patches for a range of concentrations is observed, where the strength of alignment is constant throughout the concentration range. Kinetic studies show that clustering of aligning molecules occurs with initial cluster sizes significantly below 100 µm. In the used PBLG batches with very long polymer strands of 150–350 kDa, clusters increase within minutes to diameters above 100 µm and lead to a complete phase separation in a matter of weeks. We also could show that borneol with a hydrogen bond donor hydroxyl group increases the transition concentration by approximately 0.1% wt, while camphor, without the possibility to form a hydrogen bond with PBLG, leads to a decrease of about 0.2% wt. We foresee that the transition range might be of particular interest for the measurement of residual anisotropic NMR parameters like RDCs [1,33,34,35,36] and RCSAs [11,37,38,39,40,41,42], as both isotropic and anisotropic data can be acquired simultaneously within the same sample, same volume, and identical bulk susceptibility and shim settings.

## Data Availability

All spectra acquired on a 600 MHz Bruker Avance III HD NMR spectrometer are available at the KITOpen repository at https://doi.org/10.35097/8zpe5h6qqhh5tzwn (accessed on 30 November 2025).
